# Accidental Methadone Toxicity in a 3-Year-Old Leading to Severe Cerebellitis and Death: A Case Report

**DOI:** 10.1155/crpe/1045330

**Published:** 2025-07-10

**Authors:** Jessie Pappin, Greg Hansen, Tanya Holt

**Affiliations:** ^1^Department of Pediatrics, University of Saskatchewan, Saskatoon, Canada; ^2^Department of Pediatric Critical Care, University of Saskatchewan, Saskatoon, Canada

**Keywords:** cerebellitis, leukoencephalopathy, methadone, overdose

## Abstract

Methadone is a synthetic opioid used to treat pain and opioid dependence. Despite its benefits, accidental ingestion of methadone in pediatric patients can lead to significant morbidity and mortality. Documented findings of acute toxicity secondary to methadone ingestion in children include leukoencephalopathy and cerebellitis. There is limited research into treatment and factors influencing clinical outcomes following methadone overdose in children. We report a 3-year-old child with severe brain injury and death following accidental ingestion of methadone. Our case is unique in that we were able to acquire a serum methadone level following ingestion, which was used to guide intervention and treatments. Using this case, we aim to contribute to the growing understanding of methadone toxicity while also advocating for the implementation of policies that protect our pediatric population.

## 1. Introduction

Methadone is a synthetic opioid commonly used to treat both pain and opioid dependence [1]. In Canada, access and use of methadone have drastically increased since the 1990s and have been fundamental in the treatment of opioid-use disorder [2]. Its usefulness for this purpose is related to its prolonged half-life (up to 72 h), slow onset of action, and lower rate of euphoria [3]. With methadone programs becoming increasingly common, there has unfortunately been an increase in methadone-related overdoses in the pediatric population worldwide [4].

Acute methadone toxicity in children can lead to severe morbidity and death [5]. A case review performed in 2015 outlined 58 cases of accidental pediatric methadone overdose due to improper storage of the medication. Over half of the cases described a triad of central nervous system (CNS) depression, miosis, and altered breathing pattern [6]. Alongside the clinical manifestations of opioid ingestion, there are characteristic imaging findings of leukoencephalopathy and cerebellitis reported in the literature. For example, Mills et al. describe a case of a 3-year-old with acute cerebellitis following accidental methadone ingestion [7]. In addition, Haghighi-Morad et al. conducted a literature review of accidental methadone ingestion leading to encephalopathy. They report multiple cases with findings of bilateral cerebellar damage, hippocampal damage, and cerebellitis following methadone ingestion in children [8].

In review of the case reports and literature concerning methadone overdose in children, a small cohort evolves to brain death [8]. We report a 3-year-old child with severe brain injury and death following accidental ingestion of methadone and highlight unique findings we consider important for future clinical practice and research.

## 2. Case Presentation

**Figure 1:**
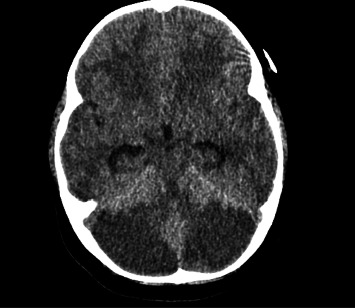
Initial head CT (12–24 h after presumed ingestion) showing diffuse edema throughout the cerebellum with no acute hemorrhage.

The patient was a 3-year-old female admitted to the pediatric intensive care unit with decreased level of consciousness and shock. Her caregiver reported that 24 h before presentation, she was difficult to rouse but quickly returned to baseline. She subsequently slept for 14 h and when her caretaker was unable to wake her, emergency services (EMSs) were notified. EMS documented a Glasgow Coma Scale (GCS) 5, poor respiratory effort, and severe hypoglycemia (1.4 mmol/L). Her hypoglycemia was promptly managed with an intravenous glucose bolus, but on arrival at the peripheral hospital, her GCS had decreased to 3 with the following vitals: BP 64/40, HR 179, O_2_ 90%, and glucose 5.1 mmol/L. She also had one episode of posturing with hypertonia. Her initial head CT revealed diffuse edema (Figure 1). She was intubated and transferred to the pediatric intensive care unit in the province's tertiary care hospital.

Upon arrival at the PICU, she was sedated and intubated. Her pupils were 2 mm bilaterally and reactive. Initial laboratory investigations were in keeping with multiorgan dysfunction syndrome (lactate of 8.5 mmol/L, creatinine of 109 μmol/L, alkaline phosphatase of 429 U/L, alanine aminotransferase of 77 U/L, aspartate aminotransferase of 268 U/L, and an INR of 1.9). At this point, the etiology of her presentation was unknown thus neuroprotective measures (head of bed at 30°–45^o^, sodium goal of 145–155 mmol/L, normocarbia, MAPs > 60, and CPP > 60) and meningitic antibiotics were initiated. She was also started on phenobarbital for seizure prophylaxis.

**Figure 2:**
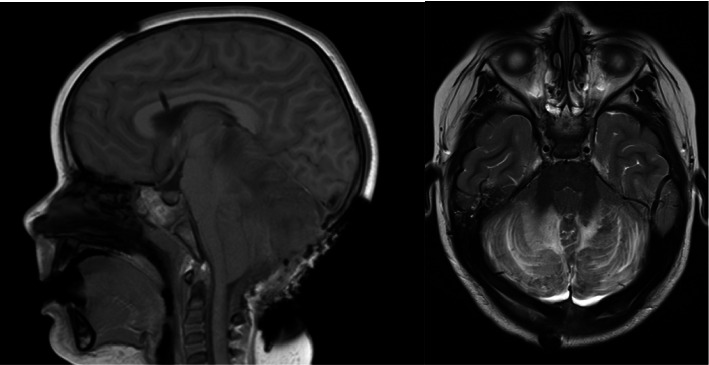
MRI (left T1 flair and right T2 TSE) status post-EVD insertion and suboccipital craniectomy. There remains severe posterior fossa mass effect owing to cytotoxic edema of the cerebellar hemispheres as described above. There are evolving supratentorial cytotoxic changes as described above, with new involvement of the globi pallidi.

On her first day of admission, urine toxicology (completed using LC/MS-MS at the provincial lab) was reported as positive for methadone. In discussion with her family, it was disclosed that there was methadone in the home prescribed to treat opioid-use disorder in her primary caregiver, and it was found opened by the patient. Unfortunately, it remains unclear what dose or formulation of methadone was ingested. Medical toxicology was consulted but naloxone was not recommended. On postadmission day (PAD) #1, magnetic resonance imaging (MRI) revealed extensive, bilateral symmetric signal changes in the cerebellum and hippocampus that are thought to represent toxic encephalopathy consistent with opioid toxicity. Downward tonsillar herniation was also evident on this MRI. Neurosurgery was consulted at this time and promptly inserted an external ventricular drain (EVD). Unfortunately, the insertion of an EVD did not improve the tonsillar herniation and neurosurgery opted for a decompressive suboccipital craniectomy (Figure 2). Following surgical intervention, she continued neuroprotective measures while intubated with little to no change in her GCS.

On PAD #2, her serum methadone was elevated at 245 nmol/L (analyzed using LC/MS-MS at the provincial lab). In collaboration with our pharmacy team, we used previous literature [9, 10] that looked at the pharmacokinetics of methadone in neonates and children following a single dose to interpret our patient's level at 48 h postingestion. Following a dose of roughly 0.06 mg/L in the study participants (presumed effective dose), their serum methadone was < 0.02 mg/L at 48 h postingestion. In contrast, our patient's serum level was calculated at 0.075 mg/L (245 nmol/L) at 48 h postingestion. This led us to believe that the initial dose was much higher than the typical effective dose for pain/sedation in children.

**Figure 3:**
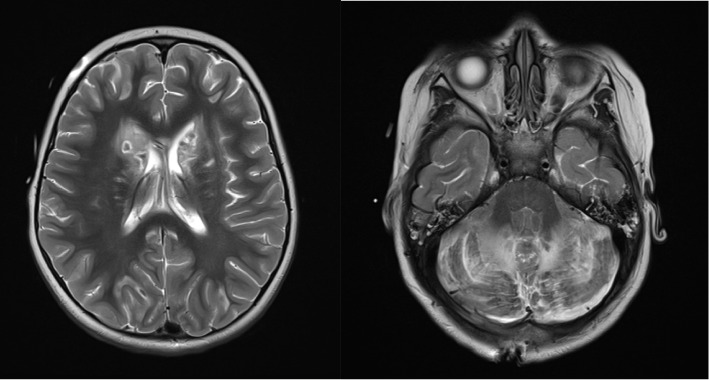
Prognostic MRI 16 days postingestion showing evolution of extensive multifocal signal abnormality. There is progressive necrosis/cavitation of the caudate heads, anterior putamen, hippocampi, and cerebellar hemispheres. Persistent diffusion restriction within the globi pallidi and new involvement of the subthalamic nuclei bilaterally.

With the lack of improvement despite various interventions and prolonged neuroprotection, a final MRI (Figure 3) was completed 16 days following ingestion which showed progression of toxic encephalopathy and severe hypoxic injury. In consultation with neurology and palliative care, the patient's family opted for comfort care and she passed away 26 days following ingestion of methadone.

## 3. Discussion

This case demonstrates the potentially devastating outcomes of methadone overdose secondary to improper storage in children despite the implementation of neuroprotective medical and surgical measures. The clinical presentation, neuroimaging results, and treatments used are consistent with previously reported cases of methadone overdose in children. However, our case is unique in that we were able to obtain both urine confirmation of methadone and its metabolites as well as a serum methadone concentration at 48 h postingestion. As mentioned above, we determined our patient's ingested dose to be high by way of comparison to previous pharmacokinetic research [9]. Knowing this, we continued neuroprotective measures for longer than is typically recommended for patients with significant brain injury.

We postulate a direct relationship between serum methadone level, severity of illness, and outcome. This is a relationship that has been demonstrated in the adult population but has yet to be established in the pediatric population. For example, a 2012 study revealed a direct correlation between serum methadone level and mortality [11]. In contrast, there has been little demonstration of this relationship in the pediatric population. A literature review on methadone toxicity in children found that of the children that died from methadone toxicity, serum concentration ranged from 60 to 1200 μg/L (0.06–0.12 mg/L) [10, 12, 13]. Although quantifiable concentrations may be helpful for risk stratification in toxic ingestions of methadone, we recognize this may be limited due to the vast interindividual variability in opioid metabolism. For example, there can be up to a 17-fold variation in methadone serum concentrations in patients receiving the same dose due to variability in the P450 enzyme system [14, 15].

Naloxone is a competitive antagonist to the μ-opioid receptor and has been a crucial antidote for opioid overdose over the years [16]. An important question that arose from this case is whether there is a clinical benefit of naloxone in toxic ingestions of methadone. Glatstein et al. (2009) completed a literature review of accidental methadone ingestions in children, including whether naloxone was used as a reversal agent. Of the cases they reviewed, over half the children were given naloxone via infusion or bolus (or both) [12]. The relationship between use of naloxone was not statistically analyzed in this study; however, none of the patients that received naloxone were declared dead [12] Aghabiklooei et al. found that in patients over 12 years of age with methadone overdose, the use of naloxone as a reversal agent did not improve mortality [17]. Further research may better define the role of naloxone in pediatric methadone overdoses.

The characteristic cerebellar and hippocampal toxic encephalopathic findings were accompanied by hypoxic injury related to delayed presentation in our case. This is synonymous with previous case reports [8] and elicits important questions related to the pathophysiologic mechanisms of methadone. Previous research has demonstrated higher quantities of μ-receptor mRNA in the cerebellum than in other areas of the brain, which could explain the characteristic neuroimaging findings that have been repeatedly illustrated in cases of overdose [18]. Demonstration of this may strengthen the argument for the use of mu-receptor antagonists such as naloxone.

In a recent report analyzing opioid therapy in Canada, they found that the number of people given opioids for opioid-use disorder increased by 44% between 2015 and 2020, with the largest increase occurring in Manitoba and Saskatchewan (287% combined) [19]. With this increase, methadone overdoses are becoming increasingly common in the pediatric population [4], therefore awareness and a high clinical index of suspicion are critical. There is a vast spectrum of clinical manifestations, use of naloxone, and outcome in these cases [8]. Our case, along with others, highlights the need for fast recognition and treatment of methadone overdose in children. It also emphasizes the need for regulation around safe storage and packaging of medications to mitigate the risk of accidental ingestion by children.

## 4. Conclusion

Our case is an example of the potential life-threatening nature of methadone ingestion in children and the importance of early recognition. Despite the considerable increase in methadone prescribing and use, there is a paucity of Canadian literature regarding methadone exposure in pediatric patients. Outside of a case report outlining intentional exposure to methadone [20], no case reports have been published outlining the potentially devastating consequences of ingestion in Canada. Furthermore, no case reports have highlighted improper storage and packaging as a cause for accidental pediatric ingestion of methadone in Canada. There is a growing need for education and policy regarding safe storage of drugs, especially those that are both widely accessible and markedly harmful if ingested by children.

## Data Availability

Data sharing is not applicable to this article as no datasets were generated or analyzed during the current study.
